# Pharmacological Actions of Multi-Target-Directed Evodiamine

**DOI:** 10.3390/molecules18021826

**Published:** 2013-01-31

**Authors:** Hui Yu, Hongwei Jin, Wuzhuang Gong, Zhanli Wang, Huaping Liang

**Affiliations:** 1 State Key Laboratory of Trauma, Burns and Combined Injury, Research Institute of Surgery, Daping Hospital, the Third Military Medical University, Chongqing 400042, China; E-Mail: huiyu2008@hotmail.com; 2 The Second Affiliated Hospital, Baotou Medical College, Baotou 014030, China; 3 State Key Laboratory of Natural and Biomimetic Drugs, Peking University Health Science Center, Beijing 100191, China; E-Mails: jinhw@bjmu.edu.cn; 4 The First Affiliated Hospital, Baotou Medical College, Baotou 014010, China; E-Mail: gongwz73@sina.com

**Keywords:** evodiamine, bioactivity, mechanism, receptor binding

## Abstract

Evodiamine, a naturally occurring indole alkaloid, is one of the main bioactive ingredients of *Evodiae fructus*. With respect to the pharmacological actions of evodiamine, more attention has been paid to beneficial effects in insults involving cancer, obesity, nociception, inflammation, cardiovascular diseases, Alzheimer's disease, infectious diseases and themoregulative effects. evodiamine has evolved a superior ability to bind various proteins, so we also argue that it is good starting point for multi-target drugs. This review is primarily addressed to the description of the recent advances in the biological activity studies of evodiamine, with a focus on pharmacological mechanism. The present review also includes the pharmacokinetics and the detailed exploration of target-binding properties of evodiamine in an attempt to provide a direction for further multi-target drug design.

## 1. Introduction

Many common diseases like diabetes, cardiovascular disease, and cancer are caused by a set of several factors, such as physiological, pathological, environmental, and lifestyle. In the past, the main effort was aimed at developing highly specific molecules acting on single targets [1]. Now, there is a general agreement that molecules interfering simultaneously with multiple targets might be more effective than single target agents [2]. Moreover, using such a multi-targeted approach could theoretically permit low-dose administration of active agents therefore reducing the potential undesired events by providing synergistic or additive preventive effects. Chinese herbal medicines are attracting intensive attention because of their history of reliable therapeutic efficacy for the prevention and treatment of various human diseases for thousands of years [3,4,5,6]. Phytochemicals from medicinal plants play a vital role in treating diseases by influencing the function of a number of diverse targets. Therefore, Chinese herbal medicines are becoming important resources for designing multi-target bioactive molecules. Accordingly, it is worth noting that genomic, proteomic, and computational approaches have been explored to identify the molecular targets of natural products. Over the past decade, more and more molecular targets of certain natural herbal product have been proposed, which is helpful for current multi-target drug discovery [7]. For example, the Zhang research group investigated the therapeutic mechanisms of astragaloside IV extracted from *Astragalus membranaceus* Bunge, a medicinal herb used for cardiovascular diseases. They identified a total of 39 putative targets of astragaloside IV, implicating that the therapeutic effects of astragaloside IV are based upon a combination of blocking calcium influx, vasodilation, anti-thrombosis, anti-oxidation, anti-inflammation and immune regulation [8].

**Figure 1:**
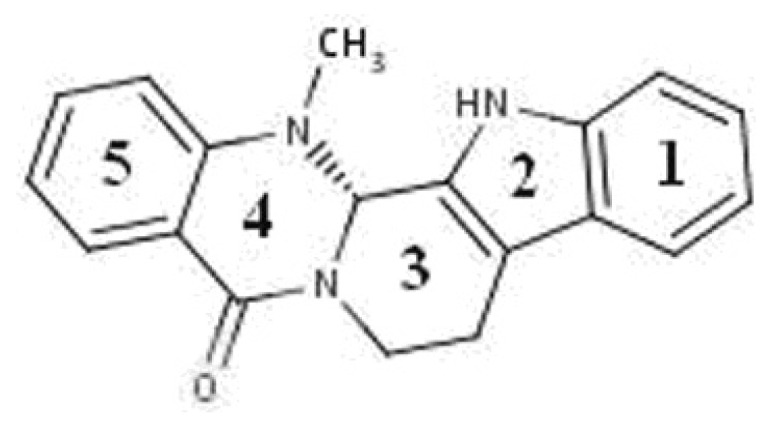
Structure of evodiamine.

The fruit of “Wu-Zhu-Yu” (*Evodiae fructus*; *Evodia rutaecarpa* Benth., Rutaceae) is one of the most *popular and* multi-purpose herbs traditionally used in China for the treatment of headaches, abdominal pain, difficult menstruation, vomiting, diarrhea, and other diseases [9]. Phytochemical studies have shown the presence of evodiamine (Figure 1), which is an indole alkaloid found in large amounts in the Chinese medicine *evodia* [10].

Growing evidence demonstrates that evodiamine represents an important compound possessing a wide spectrum of biological activities [11,12,13,14], suggesting that it might interact with a number of diverse targets to carry out its therapeutic effects. The broad spectrum of medicinal properties associated with this compound has encouraged medicinal chemists to design and synthesize a large number of novel therapeutic agents. Several analogues exhibit significant anti-tumour, anti-microbial, and anti-inflammatory activities. For this reason evodiamine is an object of continuously growing interest amongst the scientists. With the characterization of the molecular targets for evodiamine, evodiamine can be used as a promising scaffold for development of a novel class of multi-target-directed compounds, which can be beneficial for cancer or inflammatory treatment. This review attempts to summarize the recent researches on evodiamine focusing on biological activity and mechanism of action. Moreover, target-binding properties and bioavailability of evodiamine were also highlighted.

## 2. Biological Activities of Evodiamine

### 2.1. Anti-Inflammatory Activity

**Figure 2:**
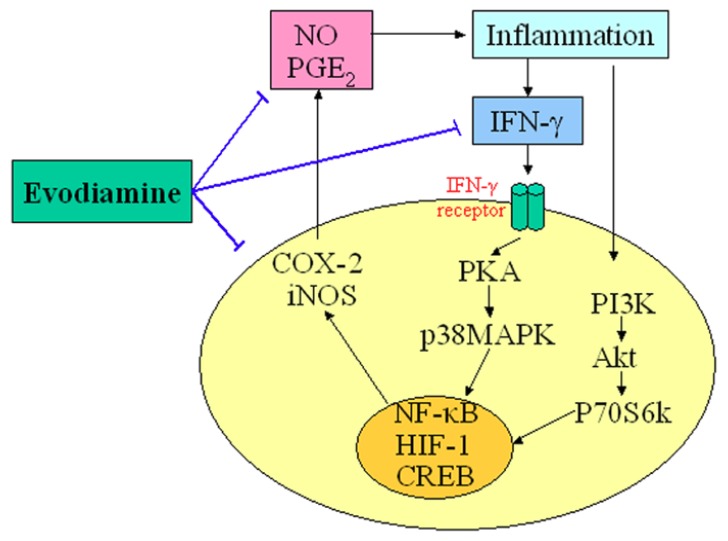
Demonstration of the key role of evodiamine in the regulation of *inflammatory*. T-lines indicate inhibitory effects.

An appreciable amount of research has reported on the potential anti-inflammatory properties and the possible underlying mechanisms of action of evodiamine. Nitric oxide (NO) is a highly reactive molecule produced from the amino acid arginine by the enzyme NO synthase (NOS). Inappropriate, excessive production of NO is largely responsible for pathogenesis of various inflammatory diseases [15]. Chiou *et al*., examined the possible anti-inflammatory effects of evodiamine by assessing its effects on NO production in cultured murine macrophage-like cell line RAW 264.7 [16]. Their results indicated that evodiamine inhibited NO production by interfering with the interferon-gamma (IFN-γ)-initiated signaling events. Ko *et al*., also found that anti-inflammatory activities of evodiamine could be partially explained by its potentials for inhibiting inducible nitric oxide synthase (iNOS)-dependent NO production in activated inflammatory cells [17]. In addition, evodiamine was found to inhibit the action of nuclear factor kappa B (NF-κB) and the transcription of cyclo-oxygenase-2 (COX-2). Liu *et al*. recently demonstrated that evodiamine inhibited COX-2 expression and hypoxia-inducible factor 1α (HIF-1α) accumulation via dephosphorylation of the serine/threonine protein kinase B (PKB/Akt) and the 70 kDa ribosomal S6 kinase (p70S6k), providing evidence for a novel mechanism underlying its anti-inflammatory activity [18]. Moreover, evodiamine has strong inhibitory effects on the synthesis of prostaglandin E_2_ (PGE_2_), which is the principal pro-inflammatory prostanoid and contributes to one of the key features of inflammation [19]. These results provide a scientific rationale for the anti-inflammatory use of evodiamine. The central role of evodiamine in the regulation of inflammatory is summarized in Figure 2.

### 2.2. Anti-Cancer Activity

**Figure 3:**
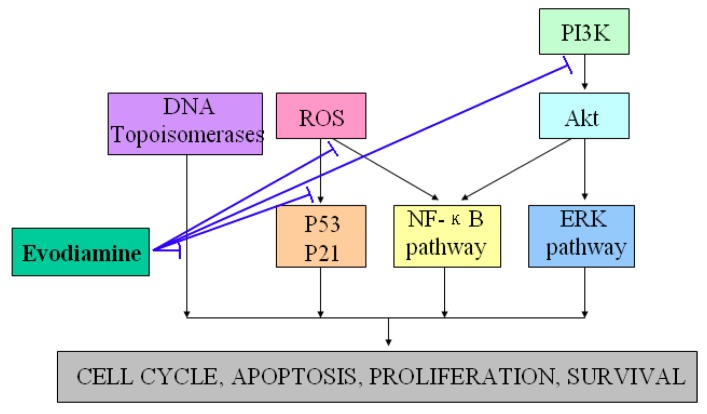
Demonstration of the *potential* anti-tumor properties and the possible underlying mechanisms of evodiamine. T-lines indicate inhibitory effects.

Evodiamine has been shown to exhibit anti-tumor properties by inhibiting proliferation of various cancer cell lines, including cervical cancer cells, colon cancer cells, lung cancer cells, melanoma cells, leukemic T-lymphocyte cells, prostate cancer cells and breast cancer cells [20,21,22,23,24,25,26,27,28]. One well-known manner of suppressing proliferation rates by evodiamine involves cell cycle progression arrest (G2/M phase) via activation of Cdc2/cyclin B [29]. In addition, evodiamine induced apoptosis of a variety of tumor cell lines through several pathways [30,31,32]. Takada *et al*. found that evodiamine exhibited apoptotic activity by modulating NF-κB activation, which leads to inhibition of NF-κB-regulated gene products such as Cyclin D1, X chromosome-linked IAP (XIAP), Bcl-2, and Bcl-Xl [33]. Other studies further revealed that evodiamine increased the expression of the apoptosis inducer Bax and decreased that of the apoptosis suppressor Bcl-2, and then induced apoptosis through the caspase pathway [34,35]. Reactive oxygen species (ROS) and NO generations were also found to exhibit regulatory effects on functions of p53, p21, protein tyrosine kinase (PTK) and other signaling proteins involved in evodiamine-induced apoptosis [36,37,38,39]. PI3K/Akt and extracellular signal-regulated kinases (ERKs) signaling pathways were also found to exhibit essential roles in the responses of tumor cells apoptosis induced by evodiamine [40,41,42,43]. Besides its anti-proliferative and anti-apoptotic effects, the effective inhibition of tumor invasion and metastasis are additional mechanisms by which evodiamine may communicate to halt the cancerous process [44]. Moreover, evodiamine has recently been identified as a dual catalytic inhibitor of topoisomerases I and II [45,46,47,48]. Figure 3 showed the potential anti-tumor properties and the possible underlying mechanisms of evodiamine.

### 2.3. Anti-Obesity Activity

Evodiamine has excellent potential as an agent to prevent obesity [49]. Kobayashi *et al*. found that evodiamine appeared to prevent obesity and reduce body fat [12]. The major mechanism eliciting the effect was postulated to be enhancement of uncoupling protein-1 (UCP1) thermogenesis through β3-adrenergic stimulation in brown adipose tissue (BAT). Shi *et al*. reported that intragastric administration of evodiamine suppressed the neuropeptide Y (NPY) mRNA and peptide levels in the arcuate nucleus (ARC) of the hypothalamus, which might be one of the mechanisms by which evodiamine exerted its fat loss effects [50]. In addition, Wang *et al*. reported that evodiamine inhibited adipogenesis by simulating the ERK/MAPK signaling pathway, which modulated the expression of the adipocyte specific transcription factors and Akt signaling [51]. They also found that evodiamine improved leptin resistance and insulin sensitivity in the mice [52]. Moreover, evodiamine inhibited both gastric emptying and gastrointestinal transit, whereas increased the plasma concentration of cholecystokinin (CCK) in a dose-dependent manner, which plays a key role in regulation of digestion and appetite [53,54].

### 2.4. Anti-Cardiovascula Disease Activity

Evodiamine showed a beneficial effect on cardiovascular diseases as reported previously [55,56,57]. Chiou *et al*., found that evodiamine had a vasodilatory effect in rat isolated mesenteric arteries and the effect was endothelium dependent [58]. Evodiamine also demonstrated significant diuretic effect due to the inhibition of aldosterone release, which can control blood volume [59,60]. In addition, several studies showed that evodiamine produced transient positive inotropic and chronotropic effects on the guinea-pig isolated atria [61]. Previous investigations also indicated that evodiamine possessed a protective effect of cardiac anaphylactic injury by stimulation of calcitonin gene-related peptide (CGRP) release [62,63,64]. Similarly, evodiamine exerted protection against myocardial ischemia-reperfusion injury in rats by activation of vanilloid receptors to stimulate the CGRP release [65,66]. Moreover, evodiamine inhibited LIGHT-induced production of chemokine receptor (CCR) 1, CCR2, intracellular adhesion molecule 1 (ICAM-1), the phosphorylation of ERKs and p38 MAPK via decreasing ROS production and NADPH oxidase activation, implicating that evodiamine has the potential for use as anti-atherosclerosis agents [67].

### 2.5. Anti-Alzheimer’s Disease Activity

Alzheimer’s disease (AD) is a progressive, irreversible brain disease caused by degeneration of synapses and death of neurons, resulting in cognitive, memory and behavioral impairments [68,69]. It is reported that a variety of biologically active constituents isolated from traditional Chinese herbs can significantly reduce the risk of AD [70,71,72,73,74,75]. Recently, the effect and the possible mechanisms of action of evodiamine in AD mouse models were investigated [76]. Evodiamine was reported to improve the cognitive abilities in the transgenic models of AD. Further investigations demonstrated that evodiamine treatment increased glucose uptake in brain tissue. The results also indicated that evodiamine inhibited the expression of COX-2 and inflammatory cytokines in AD mouse models, such as IL-1β, IL-6, and TNF-α. However, evodiamine had no effect on amyloid beta-peptide (Aβ) deposition [76]. Therefore, the effect of evodiamine on improvement of behavior in AD mouse models was likely mediated through the inhibition of the inflammatory process. Moreover, previous investigations indicated that dehydroevodiamine, an evodiamine analogue also demonstrated significant effect on AD. The chemical structure of dehydroevodiamine has been used as a basis in the clinical development of novel cholinesterase inhibitors [77].

### 2.6. Anti-Microbial Activity

Anti-microbial agents for the treatment of infections caused by bacteria, fungi and protozoa are different from the pharmacodynamic agents that affected the physiological, biochemical, or immunological function of host. The increase in number of antibiotic-resistant pathogenic bacteria has stimulated research on the development and application of new antimicrobial agents. Some compounds isolated from *Evodia rutaecarpa* were found to act as new anti-infectious agents, such as 3-dimethylallyl-4-methoxy-2-quinolone, 1-methyl-2-pentadecyl-4(1*H*)-quinolone, evocarpine, dihydro-evocarpine and 1-methyl-2-[(*Z*)-8-tridecenyl]-4-(1*H*)-quinolone [78,79,80,81,82]. Recently, Chiou *et al*. found that the compound evodiamine was effective in suppressing H1N1-induced chemokines production and blocking chemokine-attracted leukocytes recruitment, implicating potential in influenza virus infection-related inflammatory disorders [83]. Our group also found that evodiamine was capable of deactivation of *E. coli* and can be used as a natural antimicrobial agent. These results have not been reported yet.

### 2.7. Other Activities

It is well known that transient receptor potential cation channel subfamily V member 1 (TRPV1) plays a fundamental role in modulation of pain [84]. Some studies demonstrated that evodiamine exerted the analgesic effects due to its vanilloid receptor agonistic activities [85,86,87]. Evodiamine possesses many other biological functions, such as thermoregulatory effect, antianoxic action, dermatological applications, bronchoconstrictive action, and hormones secretion [88,89,90,91,92,93,94,95,96].

## 3. Protein-Ligand Interaction

Identification of molecular targets of evodiamine is an enormous opportunity for modern pharmacology. Up to data, three proteins are believed to be direct targets of evodiamine, including TRPV1, the aryl hydrocarbon receptor (AhR), and topoisomerases I and II. These proteins seem to be important in inflammation, cancer and other diseases. In fact, compounds which can regulate multiple targets may have superior utility over single-target drugs. For example, Guerrant *et al*. synthesized dual-acting histone deacetylase and topoisomerase II inhibitors, which potently inhibit the proliferation of representative cancer cell lines [97]. Recently, the structures of several complexes of evodiamine with its targets have been elucidated. Investigations of the interaction between evodiamine and various targets provided detailed information regarding the structural features required for binding, which can be exploited in future multi-target drug discovery strategies.

### 3.1. TRPV1

**Figure 4:**
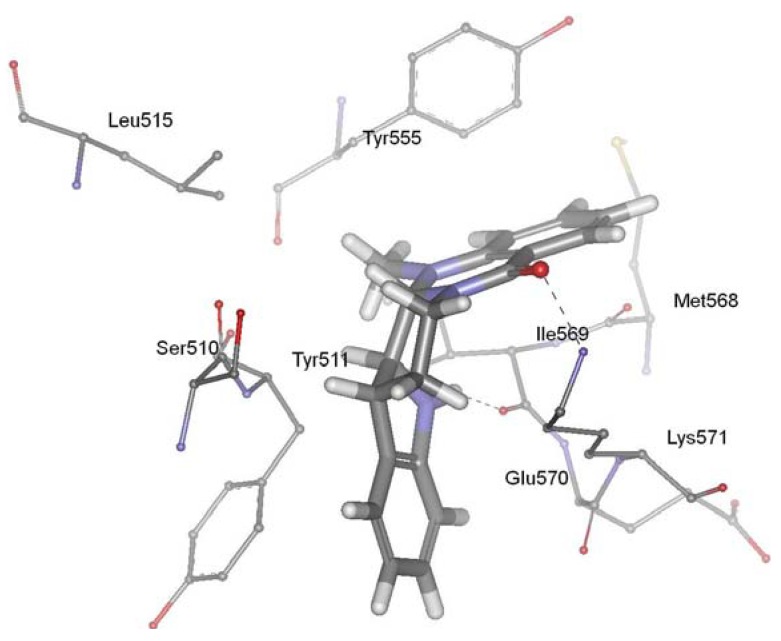
Binding mode of evodiamine in the active site of vanilloid receptor TRPV1.

TRPV1, a member of the transient receptor potential superfamily, is activated by protons, heat, endogenous substances and natural ligands such as capsaicin, resiniferatoxin, and evodiamine. The receptor participated in a wide variety of pathological and physiological processes, suggesting that regulation of this receptor activation should have considerable therapeutic utility. TRPV1 has been linked to processes mediating inflammation, cancer, cardiovascular diseases, obesity, skin diseases, and neuropathic pain [98,99,100,101,102,103,104,105,106]. TRPV1 ligands have attracted much attention as promising drug candidates to block related pathological states associated with this receptor. Evodiamine is a vanilloid receptor agonist, and thus represents a new potential class of lead molecules for new analgesics development [86]. Recently, our group investigated the precise interaction between evodiamine and TRPV1 using homology modeling, molecular docking, dynamics simulation and pharmacophore modeling methods [107]. Our results showed that the ring 1 of evodiamine pointed toward Tyr511, establishing a hydrophobic interaction. The ring 5 of evodiamine pointed toward Tyr555, forming aromatic π-π interactions. In addition, evodiamine made two H-bonds between carbonyl oxygen and amino group of Lys571 and between indol nitrogen and backbone of Ile569. Pharmacophore modeling provided further evidence for the validity of the docking studies. This study identified the structural determinants required for the interaction between TRPV1 and evodiamine, and gave new suggestions for the rational design of novel TRPV1 ligands. The biological activity experiment showed that evodiamine bound to rat TRPV1 with a Ki of 5.95 ± 0.87 microM, and yielded an EC_50_ value of 856 ± 43 nM [85]. Figure 4 showed the predicted binding mode of evodiamine in the active site of vanilloid receptor TRPV1.

### 3.2. DNA Topoisomerases

**Figure 5:**
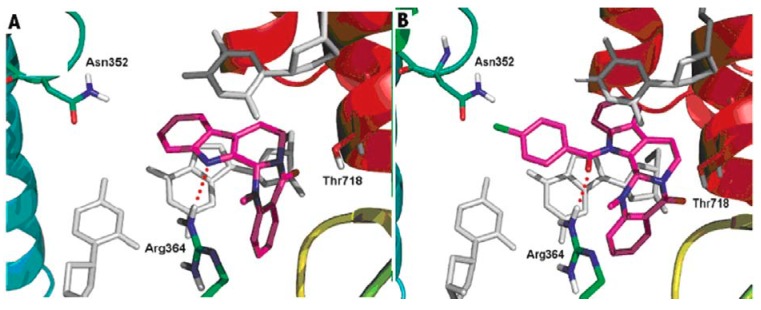
Binding modes of evodiamine and 3-(4-chlorobenzoyl) evodiamine in the active site of topoisomerase I-DNA complex.

DNA topoisomerases (I and II) are complex enzymes, which control the topological state of DNA throughout breaking and rejoining of DNA strands. Topoisomerases are involved in various DNA-related cellular processes, such as replication, transcription, recombination, chromatin condensation and daughter chromatides partitioning [108]. A lot of new data concerning basic features of the different types of topoisomerases was published. Since the structure and function were well characterized, topoisomerases have been shown to have a potential use for drug design. It is well known that the inhibitors of DNA topoisomerases showed pronounced antitumor activity [109,110,111]. Topoisomerases have been also shown to have a potential for delivering antibacterial compounds or drug candidates [112,113]. In addition, topoisomerases are involved in the cardiovascular and nervous system diseases [114,115]. Dong *et al*. gained a better understanding of the probable binding modes of evodiamine within the human topoisomerase I binding pocket. They also designed and synthesized a series of evodiamine analogues and investigated their structure-activity relationship [48]. Various groups were introduced to the indole nitrogen atom of evodiamine, and the substituted benzoyl groups were found to be favorable for the antitumor activity. The result of the biological activity test showed that the 4-Cl benzoyl derivative was the most active one with IC_50_ values in the range 0.049–2.6 μM [48]. Figure 5 shows the possible binding mode of evodiamine and 3-(4-chlorobenzoyl) evodiamine in the active site of topoisomerase I-DNA complex.

### 3.3. Aryl Hydrocarbon Receptor

**Figure 6:**
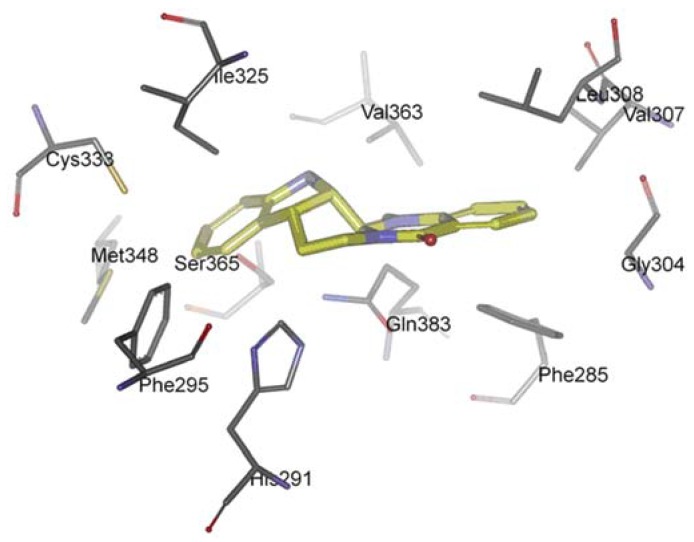
Docking orientation of evodiamine within the AhR-LBD binding pocket.

The AhR is a member of the family of basic helix-loop-helix/Per-Arnt-Sim (bHLH/PAS) transcription factors [116]. Non-ligand bound AhR is retained in the cytoplasm as an inactive protein complex bound to several co-chaperones [117,118,119]. Upon ligand binding to AhR, the chaperones are released, AhR imported into the nucleus and dimerized with AhR nuclear translocator (ARNT), leading to changes in gene transcription [120,121,122]. AhR plays a functional role in physiology and toxicology, especially in cellular proliferation and differentiation, the adaptive response, the toxic response, secretion of hormones and immunomodulation [123,124,125,126,127]. Recently, scientists found that AhR activation seems to be also important for cancer and inflammation, supporting the possibility of targeting the AhR for therapy in inflammation and a number of cancers [128,129,130]. Recently, we investigated the interaction between evodiamine and AhR [131]. We observed that a series of hydrophobic residues of AhR are involved in complex formation: Phe285, Phe295, Gly304, Val307, Leu308, Ile325, Cys333, Met348, Val363, and Ser365 (Figure 6). However, there is any hydrogen bond between AhR and evodiamine. Competitive ligand binding assay showed that evodiamine inhibited the specific binding of [^3^H]-TCDD, the best-characterised AhR agonist, to the AhR with an IC_50_ value of 44.8 ± 6.5 nM. The corresponding Ki value calculated for evodiamine was 28.4 ± 4.9 nM [131]. AhR binding studies *in vitro* provided the evidence that evodiamine was able to bind to the AhR as ligand and exhibit antagonistic effects.

## 4. Pharmacokinetics

Evodiamine is sparingly soluble. Some efforts have been done to improve the bioavailability of evodiamine. Previous studies have revealed that solid dispersions of evodiamine in hard capsules have a greater absorption rate than enriched samples of evodiamine in physical mixture hard capsules [132]. Tan *et al*. designed a novel evodiamine-phospholipid complex, which has higher bioavailability than evodiamine [133]. The pharmacokinetics of evodiamine were investigated in rats [134,135]. Recent research indicated that the plasma concentration of evodiamine reached the maximum level within 1 h after oral administration, and 19% of orally administered evodiamine was excreted in urine after 24 h. The main pharmacokinetic parameters were also calculated after oral administration of Wu-Chu-Yu extracts with different purities to rats. Additionally, Lin *et al*. developed a sensitive and selective liquid chromatography-mass spectrometry method for the determination of evodiamine in rabbit plasma for pharmacokinetic study [136].

## 5. Conclusions

This review focuses on potent and diverse bioactivities of evodiamine reported in the recent years. The target-binding properties of evodiamine were also highlighted. Information provided in this manuscript can be useful not only to exploit their biological potential appropriately, but also to develop multi-target drugs of future for treatment of various diseases.
